# Industrial machine tool component surface defect dataset

**DOI:** 10.1016/j.dib.2021.107643

**Published:** 2021-11-26

**Authors:** Tobias Schlagenhauf, Magnus Landwehr

**Affiliations:** Karlsruhe Institute of Technology, Germany

**Keywords:** Condition monitoring, Deep learning, Machine learning, Object detection, Semantic segmentation, Instance segmentation, Classification, Dataset

## Abstract

Using machine learning (ML) techniques in general and deep learning techniques in specific needs a certain amount of data often not available in large quantities in technical domains. The manual inspection of machine tool components and the manual end-of-line check of products are labor- intensive tasks in industrial applications that companies often want to automate. To automate classification processes and develop reliable and robust machine learning-based classification and wear prognostics models, one needs real-world datasets to train and test the models. The presented dataset consists of images of defects on ball screw drive spindles showing the progression of the defects on the spindle surface. The dataset is analysed via an initial object detection model available under: https://github.com/2Obe?tab=repositories. The reuse potential of the dataset lays in the development of failure detection and failure forecasting models for the purpose of condition monitoring and predictive maintenance. The dataset is available under https://doi.org/10.5445/IR/1000129520.

## Specifications Table

**Table:** 

Subject	Manufacturing Engineering
Specific subject area	The subject area is condition monitoring and lays in the intersection between the fields of Machine Learning (Computer Science) and Manufacturing Engineering/Mechanical Engineering. The subject area is of special importance for engineers who want to build intelligent and autonomous condition monitoring systems for the supervision of machine tool components.
Type of data	Image
How data were acquired	The data were acquired by a classical camera system mounted close to the system of interest during the operation of the system. Since the camera is mounted during operation different failure states are recorded which cannot be found in the literature so far. See section Experimental Design, Materials and Methods
Data format	Raw Analyzed
Parameters for data collection	For data collection a machine tool element like a ball screw drive like it is found in machine tools during industrial operation is considered.
Description of data collection	The data is collected by mounting the camera system onto the nut of the ball screw drive such that the camera looks vertically on the surface of the spindle. Hence the camera continuously records the surface of the component and is able to collect different conditions of the spindle.
Data source location	Institution: Karlsruhe Institute of Technology City/Town/Region: Karlsruhe Country: Germany
Data accessibility	Repository name: KITOpen Dataset for Defect Detection Data identification number: https://doi.org/10.5445/IR/1000129520 Direct URL to data: https://doi.org/10.5445/IR/1000129520 Dataset for Defect Classification Data identification number: https://doi.org/10.5445/IR/1000133819 Direct URL to data: https://doi.org/10.5445/IR/1000133819

## Value of the Data

• For industrial companies it is very important to keep the availability of machines as high as possible which makes it necessary to supervise the condition of machine tool components. The automation of this process saves cost and is necessary to build autonomous machines. Though, building autonomous systems requires large amounts of data showing the effects of interest. This is important because intelligent systems based on machine learning techniques need sufficient data to learn from. In the context of the automatic detection of surface defects, Cum grano salis the machine learning mode learns how images with defect and images without defect are looking. If there is not sufficient data then the model can't learn the specific characteristics. Since having data of defective components implies that a company has defective components (which is costly and should be prevented), this data is often rare in technical domains which in turn contradicts the need of large dataset for performant models.
• Especially companies developing (intelligent) condition monitoring systems for machine tool components benefit from the data. Since the availability of machines is of high importance for most industries, the dataset addresses a large circle of users.
• The dataset can be used by every company which wants to develop intelligent systems for failure detection and condition monitoring. The dataset can be used for transfer learning to enrich datasets from other technical domains supervising the condition of metallic surfaces. Examples could be the renewable energy sector e.g. to find defects on turbines or the railway sector e.g. to find defects on rails.
• The novel dataset shows image data of worn ball screw drives in a timely context.
• The dataset shows the progression of failures and delivers failures at different steps in time which is of large value for practitioners who want to detect failures as soon as possible.
• The dataset contains worn and not worn surfaces for classification. The images are annotated and the failures are provided with a segmentation mask indicating the size and location of the failures.

## 1 Data Description

### 1.1 Dataset for defect classification

The dataset is available in [1] and consists of 21853 150 × 150 pixel RGB images in the .png format showing areas with and without failures (failures are so called pitting(s)). The dataset is split such that approximately 50% of the images show pitting. Concretely, the dataset contains 11075 images without pitting and 10778 images with pitting. Each image is assigned with a label ∈ {*P*,  *N*}, where *P* stands for *pitting* and *N* stands for *no pitting*. Images followed by an underscore pursue the same logic but are turned by 90° to introduce some variance in the data. This effect can easily be reversed.

**Fig. 1:**
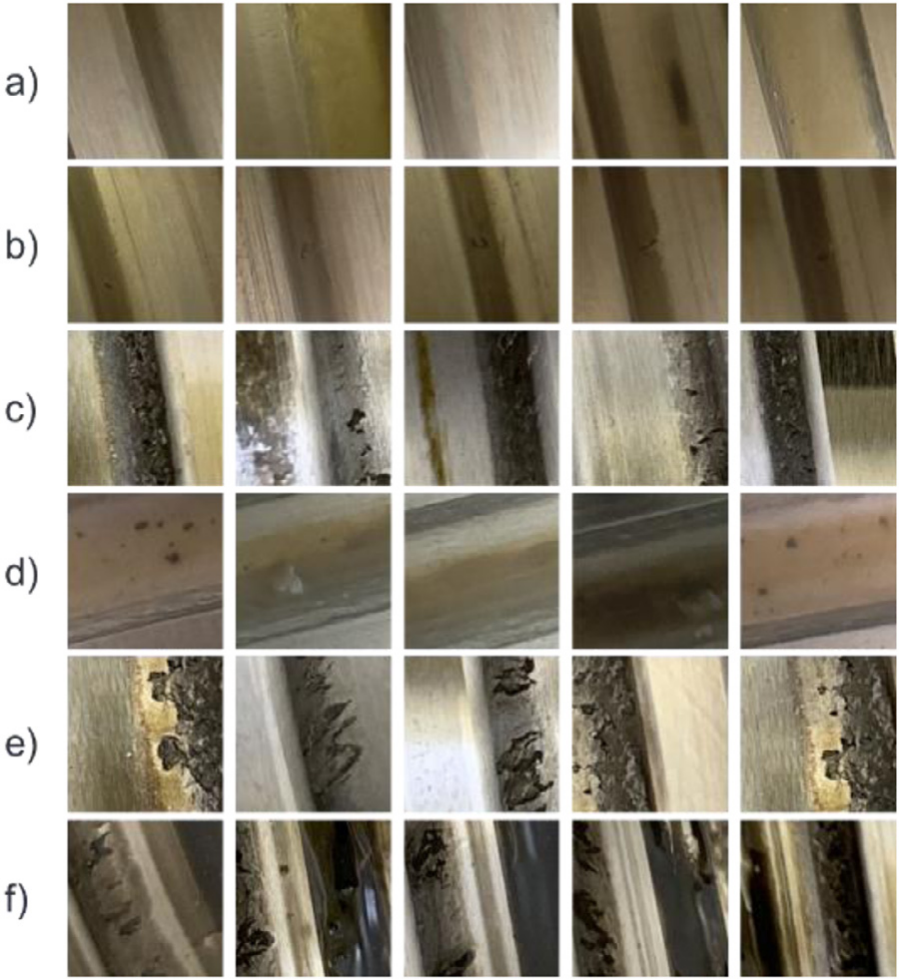
Subset of the image data taken during the destruction test.

The dataset was recorded using a camera system mounted to the ball screw drive nut as described in [2]. The camera system as well as the test procedure is depicted in Figs. 8 and 9 below. The raw data for the 150 × 150 Pixel images shown in Fig. 1 are images taken by the camera with a resolution of 2592 × 1944 Pixels, from which the images for the dataset are selected and cut out.

**Fig. 2:**
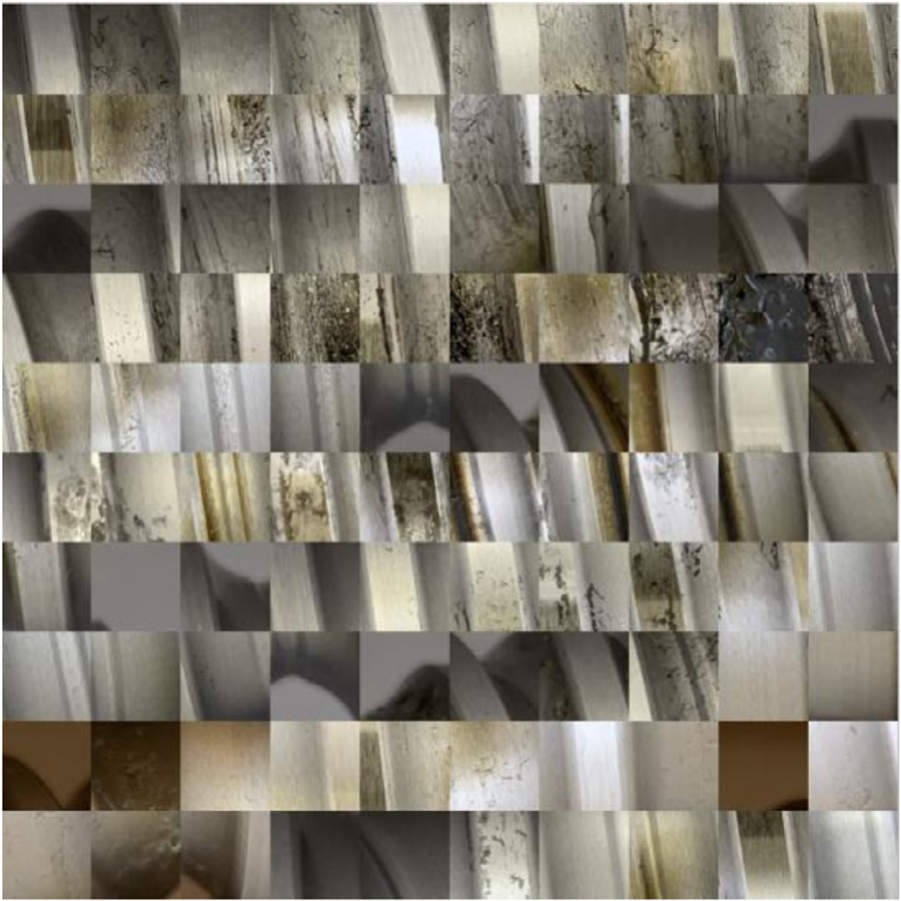
Subset of images without pitting.

**Fig. 3:**
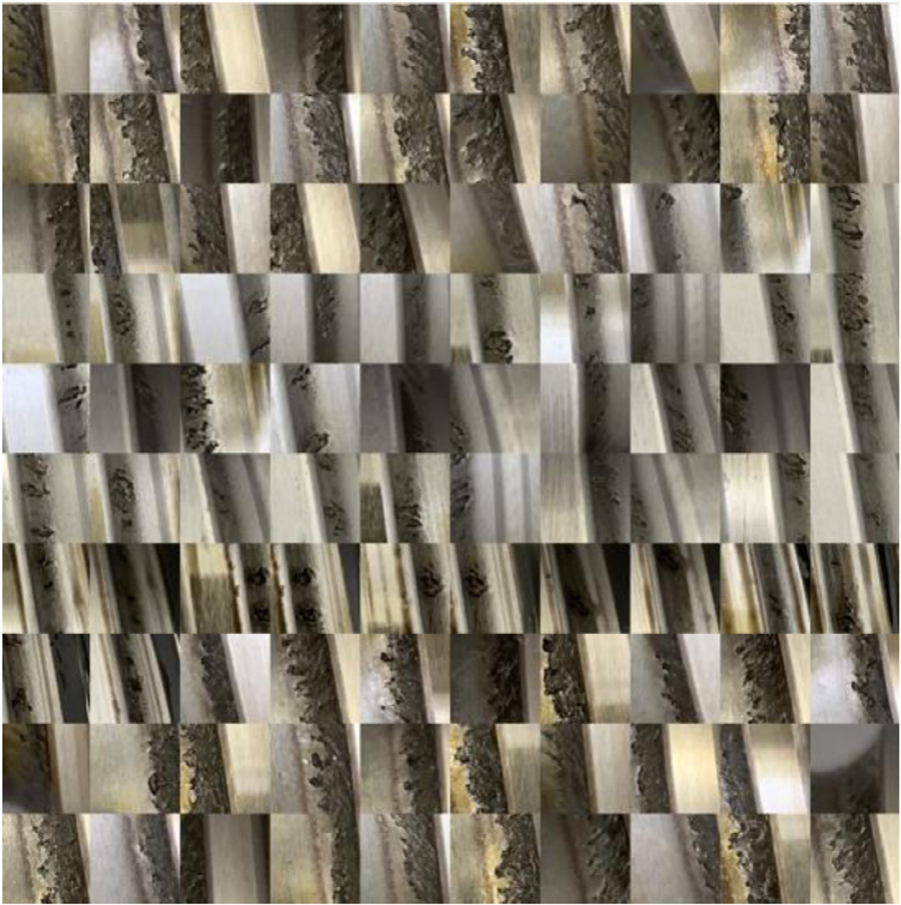
Subset of images with pitting.

The authors emphasized the selection of the images regarding the representativeness of the data. The dataset contains all sorts of conditions to which the BSDs are exposed in operation. Fig. 1 shows some representative images for the images’ whole image set. There are images showing no defect and no pollution like (a). There are images showing small pitting with no pollution (b), small pitting with pollution (c), no pitting with pollution (d), and large pitting with (e) and without (f) pollution. Hence, the whole spectrum of conditions is covered. Figs. 2 and 3 show a larger subset of images with and without pitting. It is obvious that the correct classification of images needs a substantial amount of domain knowledge.

### 1.2 Dataset for defect detection/segmentation

Besides the classification of images, the authors introduce a dataset for instance segmentation which addresses the research problem of image-based size extraction and stands out from the already available datasets for metal surface defect detection like NEU-DET [3], GC10-DET [4], or SD-saliency-900 saliency [5] with a more suitable representation of real-world problems due to containing a high-class imbalance and pixel-wise annotation masks. Furthermore, this dataset is ideally suited for application areas, namely models that are trained with little data and therefore need to have a high model efficiency.

Condition monitoring enabled by image-based size extraction to detect the current state of a machine tool element, according to [6], can, for example, lead to the reduction of equipment failure cost, improved plant reliability, and optimized maintenance intervals towards a condition-based maintenance strategy and is therefore obviously worthwhile considering. The automatic detection and evaluation of a failure is a critical step towards autonomous production machines.

**Fig. 4:**
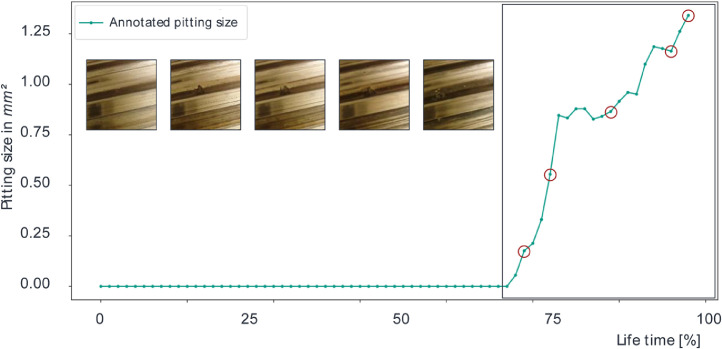
Annotated pitting size over time of a specific pitting development.

The introduced dataset is not only valuable for condition-based surface damage detection models on BSDs but also through a size progress detection on image sequences for analysis of wear development over time. This provides the community with a useful dataset for the development and test of wear detection algorithms for all machine tool elements prone to wear which can be recorded by a camera. Three important features are worth noting in particular. The dataset contains tiny damages and hence is suited to develop models especially for the detection of small, respectively early defects. In addition to that, the dataset also includes pollution origin from soil which makes detection more difficult together with foreign materials originating from e.g. the production process. As a third feature, the dataset contains the development of the same failures over a period of time. This feature can be used to develop models for the forecasting of failure progressions. To the best of our knowledge, such dataset does not exist in the literature right now. In Fig. 4, one exemplary course of an annotated size progress of the dataset is displayed.

As shown, the graph first remains for approx. 2/3 of the documented time interval at zero due to the fact that there is no surface damage. As soon as a pitting occurs, it will only continuously increase its size, in this figure represented by the pixel amount of the pitting in relation to the total pixels in an image. The drawn circles render the size of a single pitting shown in the image cutouts on the left to give an idea about the increasing pitting size. You can also see in the images increasing soiling of the surface and, therefore, there is an increasing difficulty to correctly annotate the pitting. This explains why the shown graph also contains decreasing parts, which is obviously not possible in the real application and opens the possibility to develop models able to cope with this situation.

**Fig. 5:**
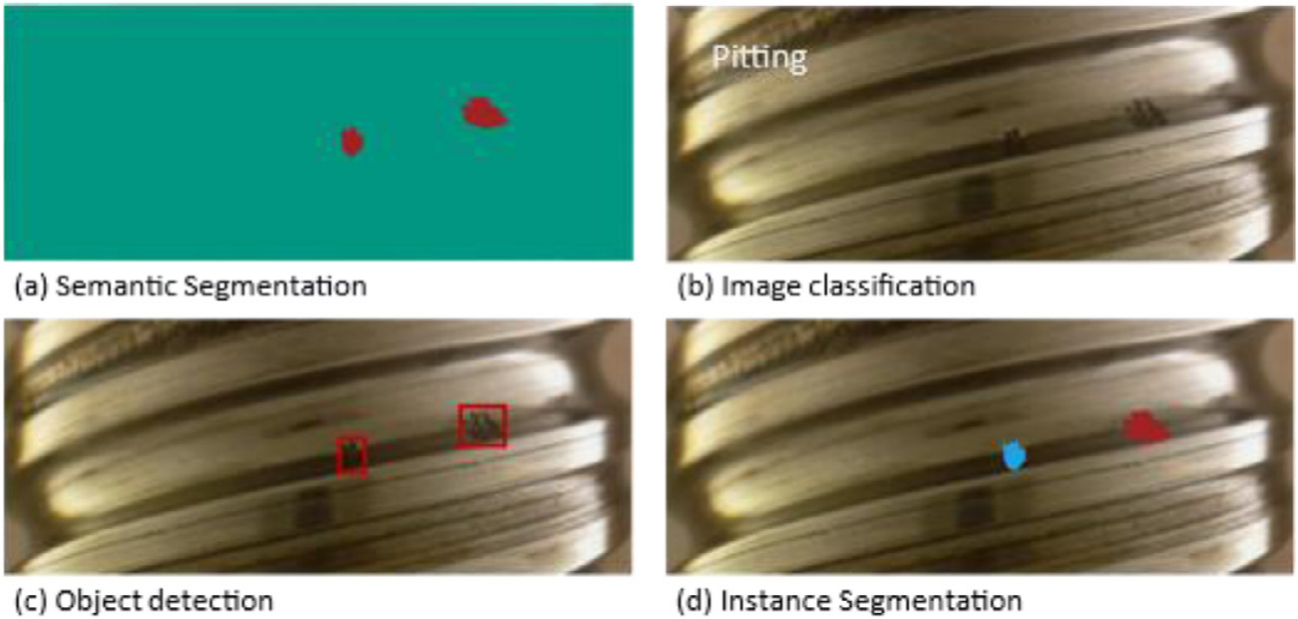
Different Image classification and Object detection types supported by the dataset.

While classification requires that its data(-points) are assigned to discrete values, such as categories [7], and detection can be used for localization of objects within images [8], it is recommendable to combine both to detect and classify single objects in images to get as close as possible to the perfect description of an image. Since these dataset annotations can be used for classification as well as detection problems, it is attainable to detect the size of an object and further, with the given wear developments, forecast the pitting size of the future. Generally, computer vision classification and detection tasks can be divided into four types (Fig. 5).

Instance segmentation (d) as for classification and detection is a pixel-wise object detection method useful for computer vision research tasks like extraction of shape and the exact size of surface damage. Known as one of the most fundamental and challenging tasks in the computer vision research area [9], this dataset can also be used for semantic segmentation (a) as a pixel-wise classification with no possibility to distinguish two or more adjacent objects from the same class, an image classification (b) for pitting recognition, and object detection (c) for single object detection.

**Fig. 6:**
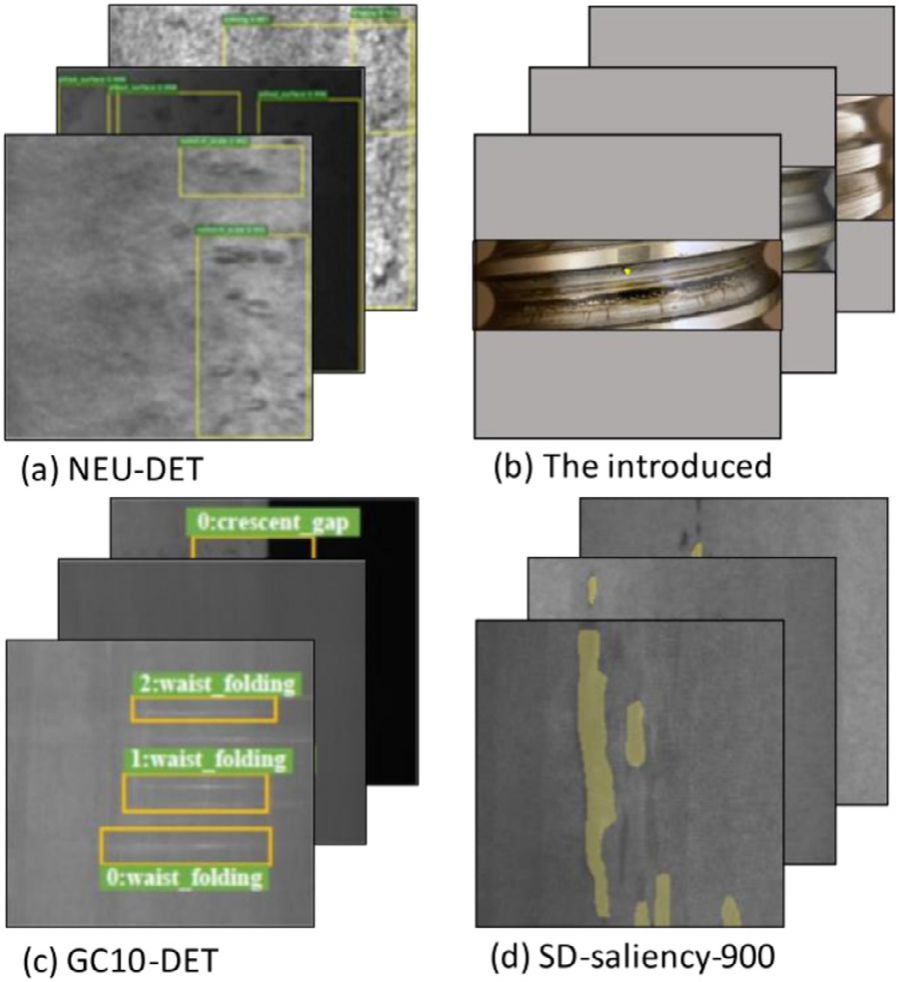
Different datasets for metal surface damage.

While most of the related research datasets for damage detection on the metal surface are not annotated for pixel-wise object detection, the introduced dataset cannot only be used for instance segmentation but moreover for the analysis of developments of surface damage over time. The (a) NEU-DET [10], shown in Fig. 6, for instance, with its 1800 200 × 200 × 1 pixel images and six annotation classes (rolled-in scale, patches, crazing, pitted surface, inclusion, scratches) or the (c) GC10-DET [4] with its 3570 2048 × 1000 × 1 big images and 10 annotation classes (cresent gap, welding line, water spots, silk spot, inclusion, oil spot, crease, punching, waist folding, rolled pit) can only be used for object detection problems.

Compared with the instance segmentation (d) SD-saliency-900 dataset [5] with its 900 200 × 200 × 1 samples, the introduced dataset contains more irrelevant surface information which is an important challenge to address since many real-world problems contain a high-class imbalance [11].

The dataset contains 1104 channel-3 images with 394 image annotations for the surface damage type “pitting”. The annotations made with the annotation tool labelme [12] are available in JSON format and hence convertible to VOC and COCO format. All images come from two BSD types.

The dataset is divided into two folders, data with all images as JPEG, labeled with all annotations, and saved_model with a baseline model. The authors also provide a python script to divide the data and labels into three different split types – “train_test_split”, which splits images into the same train and test data-split the authors used for the baseline model, “wear_dev_split”, which creates all 27 wear developments, and “type_split”, which splits the data into the occurring BSD types.

One of the two mentioned BSD types is represented with 69 images and 55 different image sizes. All images with this BSD type come either in a clean or soiled condition.

The other BSD type is shown on 325 images with two image sizes. Since all images of this type have been taken with continuous time, the degree of soiling is evolving.

Also, the dataset contains the above-mentioned 27 pitting development sequences.

**Fig. 7:**
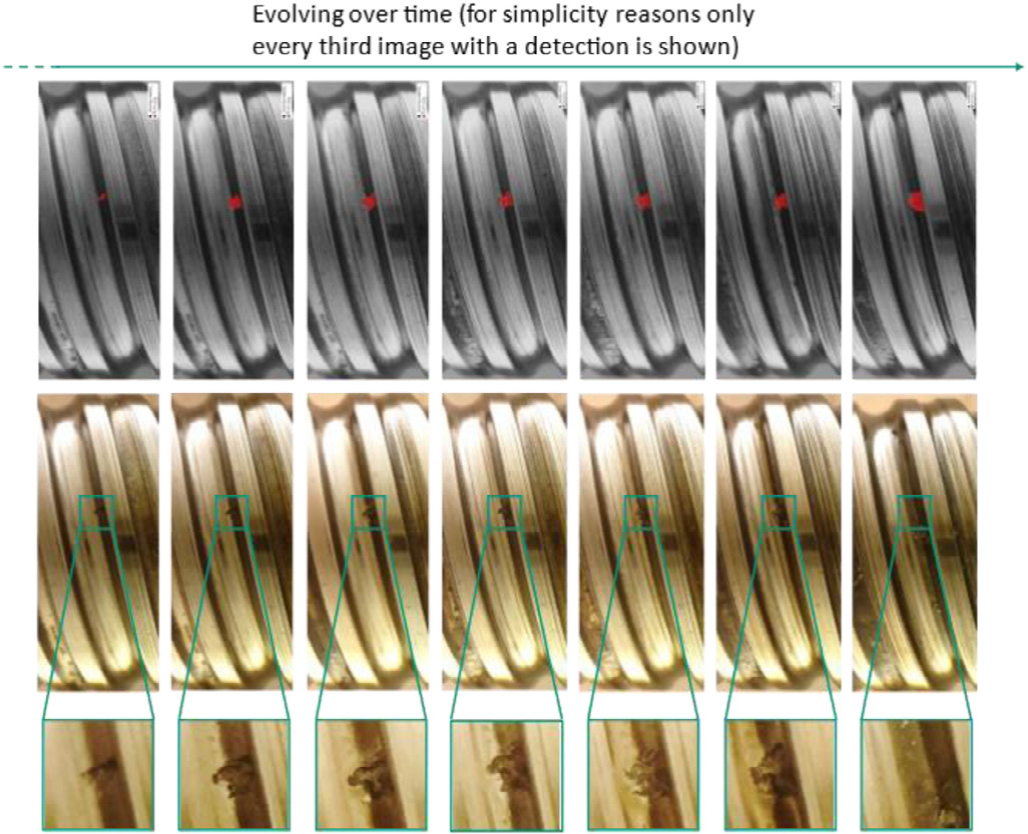
Pitting process.

Fig. 7 shows the evolving pitting development with and without the shown annotations from one of the 27 pitting developments. For convenience, only every third image starting at the beginning of the pitting formation is displayed.

## 2 Experimental Design, Materials and Methods

### 2.1 Sensor system

**Fig. 8:**
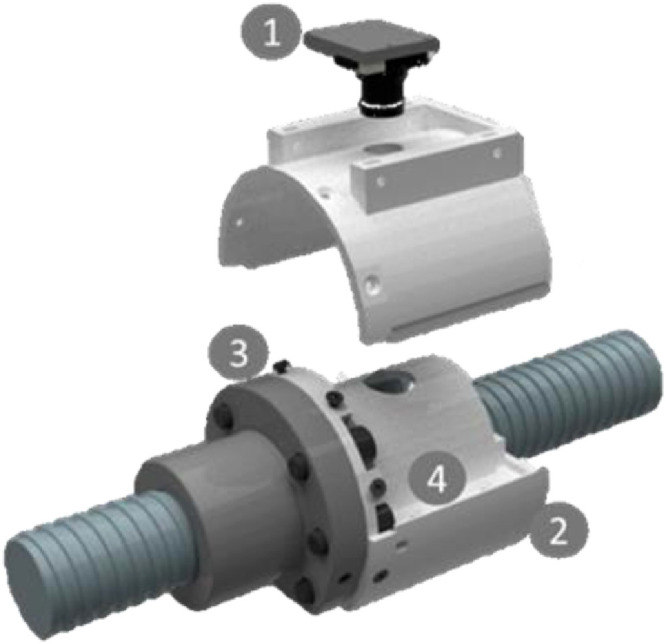
Sensor system used for image generation.

The sensor system used for the creation of the image dataset is depicted in Fig. 8.

The system is mounted onto the nut of the BSD using a mounting adapter numbered with #3. The camera (#1) looks through a hole in the so-called diffusor (#4) onto the spindle. Since turning the spindle leads to a linear motion of the nut and the spindle is turning underneath, the camera gets to see all raceways of the spindle. Using this setup, the whole spindle can be photographed. #2 is a manufactured housing enclosing the spindle which is used to ensure uniform lighting conditions during the experiment. Additionally, the housing protects the camera from pollution. An important part of the system which is responsible for lightning of the images is the so-called diffusor which also implements the light sources. The light sources are two standards LED stripes mounted onto the surface where #4 is located. The diffusor itself is 3D- printed and consists of a semitransparent plastic leading to diffuse light. Since the LEDs are not pointed onto the spindle but directly onto the housing, the light does not get directly onto the spindle but is reflected by the housing and then further made more diffuse bypassing the diffusor. During tests, this setup was found to be yielding the best results for our purpose. The used camera system is a standard Raspberry Pi V2 microcontroller camera which is a good tradeoff between resolution, costs, and necessary mounting space. The camera is set up to take images with a resolution of 2592 × 1944 pixels per image.

### 2.2 Test setup

**Fig. 9:**
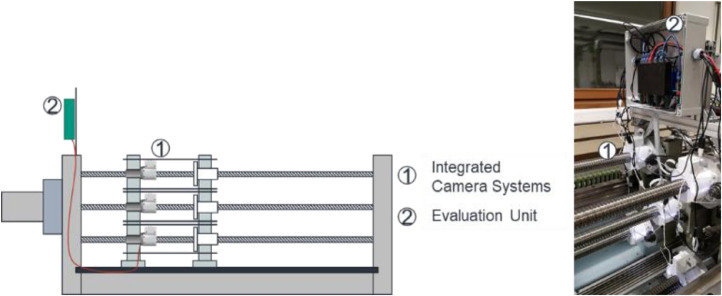
Test bench with mounted camera systems for image generation.

The dataset is generated on a test bench located at the Institute of Production Science at the Karlsruhe Institute of Technology. The test bench is depicted together with the mounted camera systems in Fig. 9.

The test bench is constructed such that a maximum of five spindles can be worn in parallel. The spindles are positioned like the five on a dice, with the middle spindle being the leading spindle connected to the motor. The other four spindles are operated by a chain drive connected to the central spindle, thus it is ensured that all spindles are operated in the same way. The spindles used are standard 32mm diameter spindles with no special treatment or prestress. Each spindle is preloaded with 70% of the *C_a_* given by the manufacturer, where 100% of the *C_a_* is the axial load at which the manufacturer ensures a safe operation of 10^6^ revolutions. In this case, the *C_a_* is chosen with 12kN. With this setup, the camera automatically triggers a complete surface recording every four hours. Between each image, the spindle is turned by an additional 22.5°, and an area of 150 × 150 pixels is cropped automatically from the large image.

### 2.3 Data analysis baseline

Regarding the introduced dataset, the authors also present a baseline model. The here used model architecture is a Mask R-CNN (regional Convolutional Network) [13] with an on the COCO dataset [14] pretrained Inception ResNet v2 [15]. The Mask R-CNN architecture is composed of two stages, a faster R-CNN with a deep convolutional network composed of Inception v4 and ResNet building blocks united in an Inception ResNet v2 architecture and an FCN (fully convolutional network). Here the authors used the standard implementation as used in [13]. For further implementation details please consider this source.

**Fig. 10:**
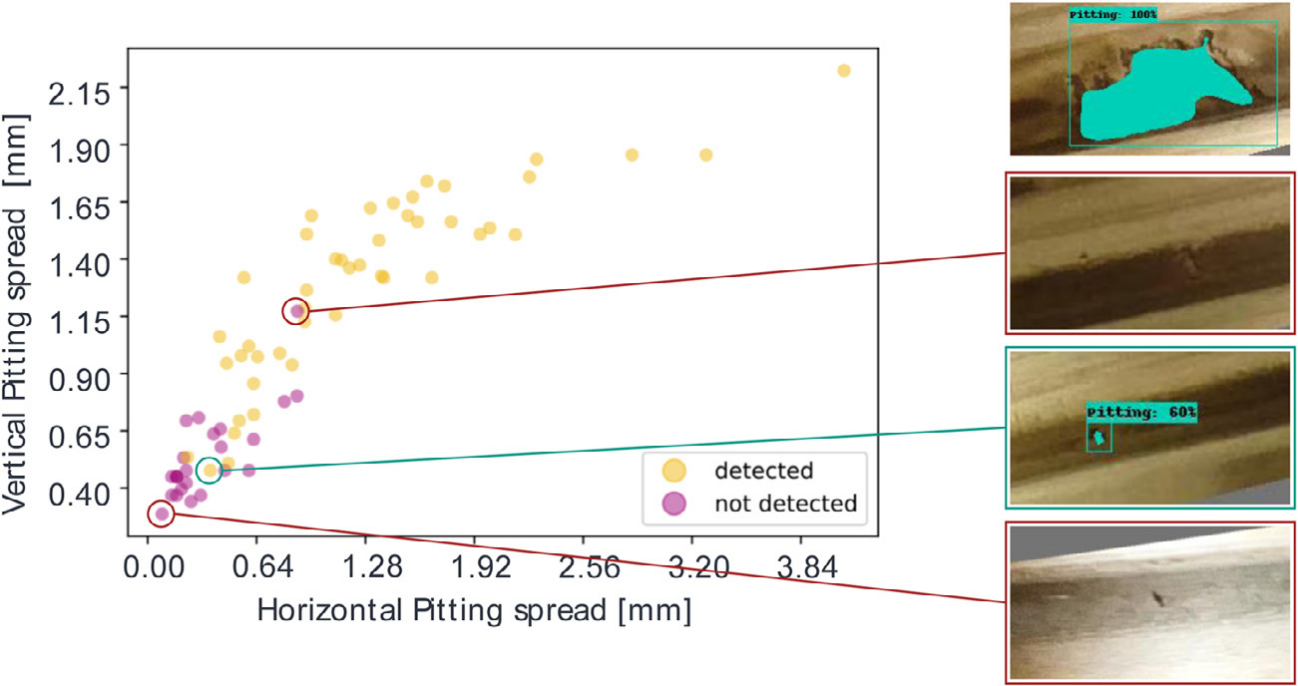
Relationship between pitting detection and its relative size.

**Fig. 11:**
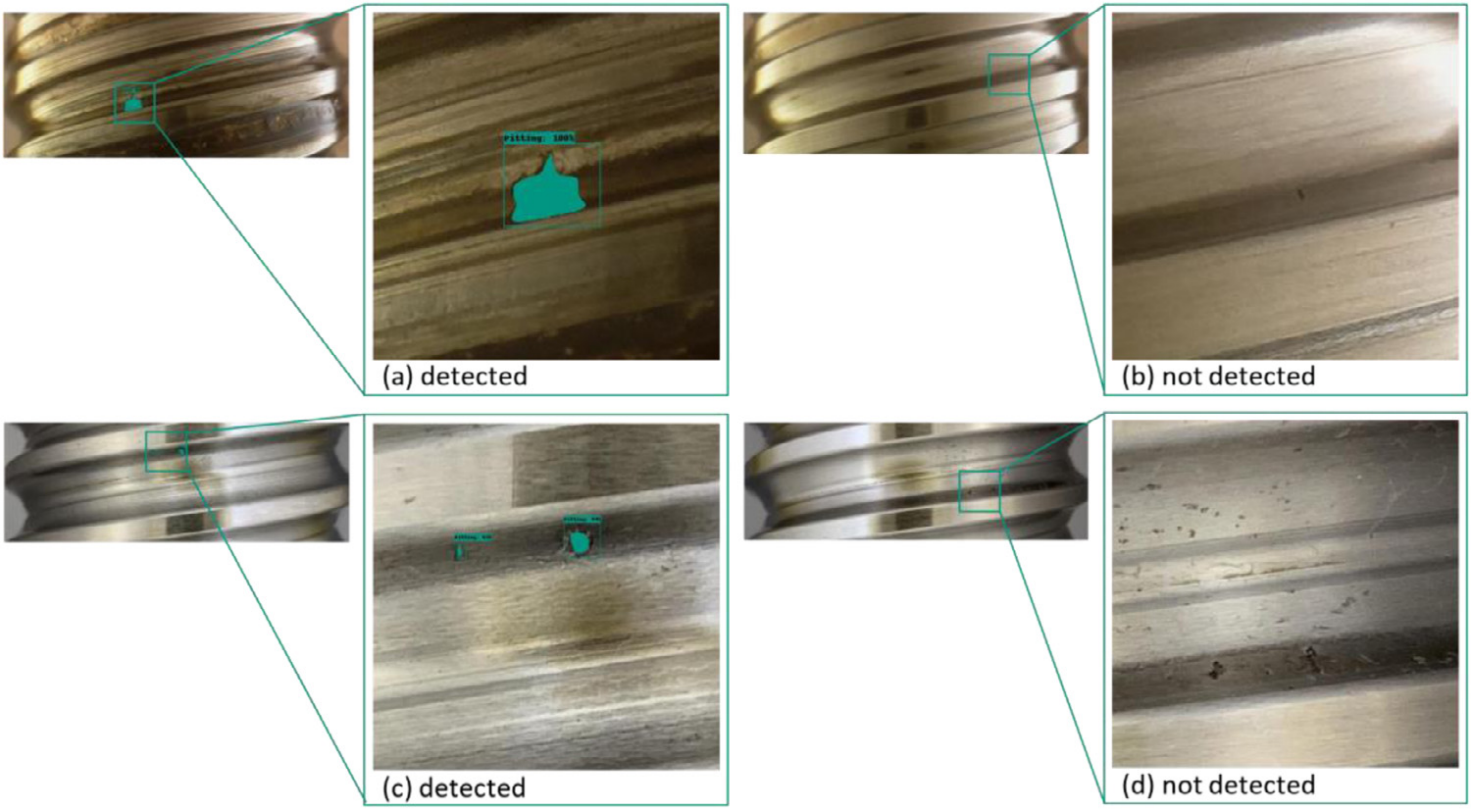
Prediction examples from the author's model.

With the chosen architecture, the authors achieved a mIoU (mean intersection over union) baseline score of 0.316. It is noticeable that the model has difficulties predicting small pitting in general (Figs. 10 and 11).

Examining the horizontal and vertical development of pitting and relating it to a binarized model prediction, a zero-one principle - where zero corresponds to “not detected”, we can see that pitting detection becomes more reliable as development increases. In Fig. 10, the circumstance just described can be readily understood. The relative horizontal spread of the pitting (width) is described on the x-axis and the relative vertical spread (height) is described on the y-axis. The binarization of the detection is represented by the coloring of the points. Fig. 11 visualizes the just mentioned circumstance on selected examples.

The pitting shown in image cutout (a) was due to its large horizontal and vertical spread detected. While the not detected pitting in cutouts (b), (d), and the detected pitting in (c) are relatively small. For convenience, the trained model will be provided. The code for the baseline detection model is available under: https://github.com/2Obe/BSData.

## Ethics Statement

The authors read and follow the ethical duties of authors.

## CRediT authorship contribution statement

**Tobias Schlagenhauf:** Conceptualization, Methodology, Data curation, Software, Writing – original draft. **Magnus Landwehr:** Data curation, Software, Writing – original draft.

## Declaration of Competing Interest

This work was supported by the German Research Foundation (DFG) under Grant FL 197/77-1. The authors further declare that they have no competing interests.

The authors declare that they have no known competing financial interests or personal relationships which have or could be perceived to have influenced the work reported in this article.
